# Reparameterization of GFN1-xTB for atmospheric molecular clusters: applications to multi-acid–multi-base systems†

**DOI:** 10.1039/d4ra03021d

**Published:** 2024-06-21

**Authors:** Yosef Knattrup, Jakub Kubečka, Haide Wu, Frank Jensen, Jonas Elm

**Affiliations:** a Department of Chemistry, Aarhus University Langelandsgade 140, Aarhus C 8000 Denmark jelm@chem.au.dk +45 28938085

## Abstract

Atmospheric molecular clusters, the onset of secondary aerosol formation, are a major part of the current uncertainty in modern climate models. Quantum chemical (QC) methods are usually employed in a funneling approach to identify the lowest free energy cluster structures. However, the funneling approach highly depends on the accuracy of low-cost methods to ensure that important low-lying minima are not missed. Here we present a reparameterized GFN1-xTB model based on the clusteromics I–V datasets for studying atmospheric molecular clusters (AMC), denoted AMC-xTB. The AMC-xTB model reduces the mean of electronic binding energy errors from 7–11.8 kcal mol^−1^ to roughly 0 kcal mol^−1^ and the root mean square deviation from 7.6–12.3 kcal mol^−1^ to 0.81–1.45 kcal mol^−1^. In addition, the minimum structures obtained with AMC-xTB are closer to the ωB97X-D/6-31++G(d,p) level of theory compared to GFN1-xTB. We employ the new parameterization in two new configurational sampling workflows that include an additional meta-dynamics sampling step using CREST with the AMC-xTB model. The first workflow, denoted the “independent workflow”, is a commonly used funneling approach with an additional CREST step, and the second, the “improvement workflow”, is where the best configuration currently known in the literature is improved with a CREST + AMC-xTB step. Testing the new workflow we find configurations lower in free energy for all the literature clusters with the largest improvement being up to 21 kcal mol^−1^. Lastly, by employing the improvement workflow we massively screened 288 new multi-acid–multi-base clusters containing up to 8 different species. For these new multi-acid–multi-base cluster systems we observe that the improvement workflow finds configurations lower in free energy for 245 out of 288 (85.1%) cluster structures. Most of the improvements are within 2 kcal mol^−1^, but we see improvements up to 8.3 kcal mol^−1^. Hence, we can recommend this new workflow based on the AMC-xTB model for future studies on atmospheric molecular clusters.

## Abstract 2

Reparameterization of GFN1-xTB for atmospheric molecular clusters leads to a massive decrease in energy errors and deviation.

## Graphical abstract

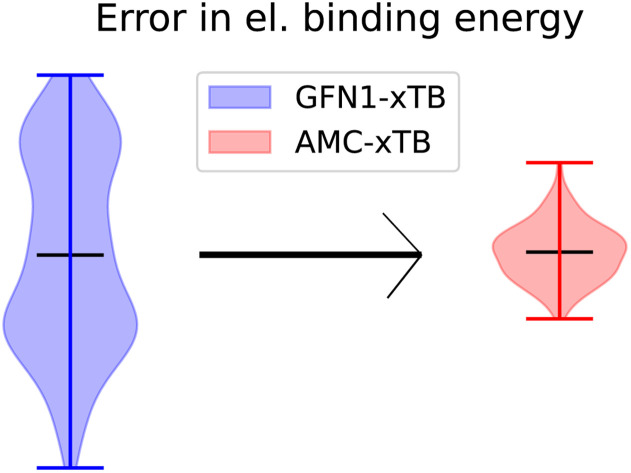

## 1 Introduction

Molecular clusters, formed through the aggregation of various atmospheric species, play a central role in aerosol particle formation.^1^ Aerosols are liquid or solid fine particles suspended in air that can act as cloud condensation nuclei (CCN) if they reach sizes at or above 50–100 nm.^2^ Roughly 50% of CCN are believed to be initially formed as clusters.^3^ CCN acts as nucleation cores for water uptake and then further growth into clouds meaning there is a direct correlation between aerosols and cloud number/properties and hence the climate. The biggest uncertainty in modern climate forcing predictions is due to the uncertainties from aerosol–cloud interactions.^1^

Sulfuric acid has been shown to be the main driver of cluster formation. Other key species are believed to be, bases (ammonia and amines), acids (methanesulfonic acid, nitric acid, iodine acids or organic acids), highly oxygenated organic molecules and water.^4–7^ It is extremely difficult to experimentally measure the composition and formation mechanism of the initial clusters due to their small size and neutral charge. Mass spectrometry techniques can measure the cluster compositions of the charged cluster, however, it is unknown if the ionization of neutral clusters significantly changes the cluster composition/structure and it is also believed that fragmentation may happen in the instruments.^8–10^ This leaves theoretical studies as the only way to elucidate the thermodynamics, kinetics, and molecular interactions governing cluster formation and its evolution. The main challenge for studying atmospheric molecular clusters is their complex configurational spaces, which require advanced configurational sampling techniques and computationally demanding quantum chemistry methods to evaluate the cluster properties accurately.^5^ Furthermore, atmospheric clustering is believed to be a multi-species process,^11^ adding another dimension of chemical complexity.

Thoroughly exploring the configurational space of atmospheric molecular clusters using, for instance, metadynamics simulations^12,13^ or genetic algorithms^14–16^ at a high level of theory is extremely computationally demanding. Hence, usually, a funneling approach^5,7,17^ is applied, where the configurational space is initially explored at a low level of theory such as force-field or semiempirical methods, and only a subset of low energy structures is selected, reoptimized, and reexamined at a higher level of theory. This process is repeated with an increasing level of theory until only a few structures remain for evaluation at the desired high level. Schematically, the process can be given as:

(1) Generate initial cluster configurations:

     ABCluster/OGOLEM/Basin hopping or similar.

(2) Semi-empirical calculations:

     Optimization at the PM6/PM7/GFN1-xTB/GFN2-xTB or similar level.

(3) DFT calculations:

     DFT optimization and vibrational frequency calculations.

(4) Single point energy refinement:

     Single-point energy calculation at coupled cluster level on the DFT optimized geometry.

Between each step in the funneling approach, filtering can be applied to reduce the number of structures that need to be handled. This can either be based on an energy threshold or a set number of cluster structures. Eventually, we end up with a handful of structures at the highest obtainable level.

The first step in the funneling procedure is the generation of a large number of configuration candidates. The key idea is sampling a large part of the potential energy surfaces at a low level of theory to get estimates for the global free energy minimum. This is usually carried out using force-field methods in combination with genetic algorithms such as in ABCluster^15,16,18–21^ and OGOLEM,^14,22–27^ by random/manual sampling or using dynamic methods such as basin hopping.^28–32^ The major issue at this step is that most force-field methods are unable to describe bond-breaking, such as proton transfer reactions, which are important for atmospherically relevant molecular clusters, requiring the sampling to include monomers where the hydrogens have been transferred to get adequate sampling. Furthermore, the accuracy of force-field methods is also insufficient to determine a subsample of the conformer candidates and all the candidates have to be taken to the higher level of theory.

The next step is semi-empirical calculations as these are a better description of the chemistry and filtering can be applied. Of the common semiempirical methods, GFN1-xTB,^33^ GFN2-xTB,^34^ PM6,^35^ and PM7 ^36^ are the most used in configurational sampling procedures for atmospheric molecular clusters.^5–7^ GFN stands for geometries, frequencies and non-covalent interactions, which are the main target properties for the method. PM stands for parameterization method indicating the model version. Of these methods, GFN1-xTB has shown to have the highest correlation with electronic binding energies at a higher level of theories^37,38^ and have been shown to have a higher correlation with DFT trajectories for molecular dynamics than GFN2-xTB.^39^ The reason GFN2-xTB performs worse than GFN1-xTB for atmospheric molecular clusters (often involving sulfuric acid) is that there is a decrease in the number of d-functions for sulfur in the basis set for the newer GFN2-xTB model.

The third step is the subsequent optimization and vibrational frequency calculation of the structures with DFT. This is the main bottleneck in the sampling methodology as limited computational resources only allow a fixed number of DFT structures to be optimized. Therefore some form of filtering is required, often based on structural properties or electronic energies from the semiempirical calculations. To circumvent the inaccuracies of semiempirical methods an intermediate step can be included, involving single-point energy calculations at the DFT level on as many structures as possible. Another option for an intermediate step is the utilization of machine learning (ML) methods. One can calculate a subset of the structures at a desired DFT level and train an ML model to predict the energies of the remaining structures.^39,40^ However, to mimic accurate DFT energies, kernel-based ML methods become computationally demanding^40–42^ and neural-networks will require an extensive set of training data and hyperparameter optimization.^43^ Moreover, ML methods often fail when predicting on structures different from the training set.

Overall, the funneling approach is never more efficient than its weakest link given by the semiempirical step in 2, in which accuracy determines the number of structures that have to be optimized/have single points calculated at the DFT level. In this paper, we focus on reparameterizing the GFN1-xTB method based on DFT energies of atmospherically relevant molecular clusters yielding a GFN1-xTB model reparametrized based on ωB97X-D/6-31++G(d,p) for ‘atmospheric molecular clusters’ denoted AMC-xTB. This new parameterization is used to sample 288 large multi-acid–multi-base clusters containing AM equivalent to the clusters studied by Knattrup *et al.*^44^

## 2 Methodology

### 2.1 Computational details

Single-point energies, gradients, and geometries for the reparameterization, configurational sampling, and comparisons were calculated using the xtb 6.4.0 program using the GFN1-xTB^33^ and AMC-xTB parameterizations. A modified version of ArbAlign^45^ available in the JKCS program^46^ was used to calculate the root-mean-square differences (RMSD) between molecular structures. Gaussian 16, version B.01 ^47^ was used for the DFT calculations. CREST 2.12 ^12,13^ with an energy window of 15 kcal mol^−1^ and in noncovalent interaction mode and ABCluster 2.0 ^15,16^ with a population of SN = 3000, number of generations of *g*_max_ = 200, and gen. survival of *g*_limit_ = 4 were used for additional configurational sampling.

### 2.2 Cluster data sets

For reparameterization of GFN1-xTB, we used the clusteromics I–V data sets^48–52^ containing (acid)_0–2_(base)_0–2_ clusters of the following atmospherically relevant species: sulfuric acid (SA), methanesulfonic acid (MSA), nitric acid (NA), formic acid (FA), ammonia (AM), methylamine (MA), dimethylamine (DMA), trimethylamine (TMA) and ethylenediamine (EDA). All structures are optimized at the ωB97X-D/6-31++G(d,p) level of theory, as benchmark studies^37,53^ show this to be a good compromise between accuracy and speed, and we used up to the three lowest electronic energy configurations found per each cluster as the optimization set for GFN1-xTB reparametrization. This leads to an optimization set comprising of a total of 1073 clusters. The GFN1-xTB reparameterization based on this optimization set will be denoted as the AMC-xTB model.

All new data calculated is freely available in the Atmospheric Cluster DataBase^54^ along with the new AMC-xTB parameter file (see Section S2).†

### 2.3 Optimization strategy

The GFN1-xTB model contains 15 global parameters, 2 element-pair-specific parameters, and 32 element-specific parameters of relevance to the species present in the optimization sets (H, C, N, O and S atoms). Initially, the Hessian was generated to probe the sensitivity of the parameters, however, we found it computationally feasible to employ a similar optimization strategy to the original GFN1-xTB paper,^33^ where we optimize all relevant parameters simultaneously. We utilize a modified version of an in-house pseudo-Newton–Raphson optimizer by Jensen *et al.*^55^ for the optimization of a target function (*T*) containing a linear combination of the difference in electronic binding energies (Δ*E*^b^) in kcal mol^−1^ and gradient norms (*g*^norm^) in hartree bohr^−1^ radius at the current GFN1-xTB parameterization and the target ωB97X-D/6-31++G(d,p) level of theory:1
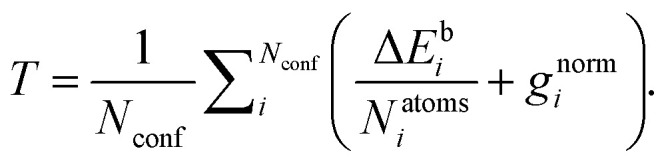
here, *N*_conf_ is the total number of structures in the optimization set, *N*^atoms^_*i*_ is the number of atoms in the *i*-th structure. We normalized by the number of atoms in each structure to prevent “overfitting” to the larger clusters.

We chose the electronic binding energies as the target properties to get a better tool for filtering based on energies in configurational sampling procedures. The gradients were included directly in the target function. We use equilibrium structures at the given level of theory, which are supposed to have near-zero gradients. However, the upper limit is set by the accuracy threshold within the xTB program during the optimization, which makes the gradients non-zero in the calculations.

Including only the electronic bindings energies in the target function yields a much better fit for the energies but causes the gradients to “explode”, effectively rendering the optimization functionality of AMC-xTB useless for our target species. Giving higher weight to the gradient norms in the target function makes the optimized structures more similar to the target ωB97X-D/6-31++G(d,p) level of theory, however, we found that it causes problems in the configurational sampling procedure where the decreased accuracy in determining the binding energies causes our configurational sampling to yield high-energy conformers at the DFT level. Overall, we found that including the gradient norms and difference in electronic bindings energies in a 1 : 1 ratio as the best compromise between the two properties.

### 2.4 Updated configurational sampling workflows

The strength of the new AMC-xTB model is that it can be used directly in configurational sampling programs such as ABCluster and CREST. Here, we will test two different new workflows for applying the reparameterized models in cluster configurational sampling.

#### 2.4.1 Original workflow

The workflow usually employed in studying atmospheric molecular clusters can be summarized as:ABCluster → GFN1-xTB^opt^_all_ → DFT^opt^_*N* lowest_here the number of configurations *N* that have to be optimized at the DFT level is a severe bottleneck in the number of new cluster systems that can be studied. Usually, 50–100 configurations are optimized at the DFT level.

#### 2.4.2 Independent workflow

The independent workflow refers to configurational sampling from scratch using the well-established funneling approach using AMC-xTB instead of GFN1-xTB.^5,17^ As the aim for the approach is to be generally applicable, we also included an additional CREST step, as it should be better at handling flexible organic compounds:ABCluster → AMC-xTB^opt^_all_ → CREST(AMC-xTB)_1 lowest_ → DFT^opt^_50 lowest_here, ABCluster, a genetic algorithm for sampling clusters, is used for the initial sampling of all possible neutral/ionic combinations of monomers that yield overall neutral clusters. The xTB 6.4.0 program was then used to optimize all the configurations at the AMC-xTB level. The cluster lowest in electronic energy was then taken as the input structure for CREST in non-covalent interaction mode, again using our new AMC-xTB model. The initial ABCluster sampling is needed because we found CREST to be quite sensitive to the input structure and, therefore, needs a good guess for a starting structure. The 50 structures lowest in electronic energy are then optimized at the DFT level.

#### 2.4.3 Improvement workflow

The improvement workflow refers to using the best structure currently known at the corresponding level of theory as the input structure for CREST using the AMC-xTB model.Best structure → CREST(AMC-xTB) → DFT^opt^_50 lowest_from here on, the workflow is the same as the independent workflow, where the 50 structures lowest in electronic energy are optimized at the corresponding DFT level.

## 3 Results and discussion

### 3.1 Extension of the multi-acid–multi-base dataset

**Fig. 1:**
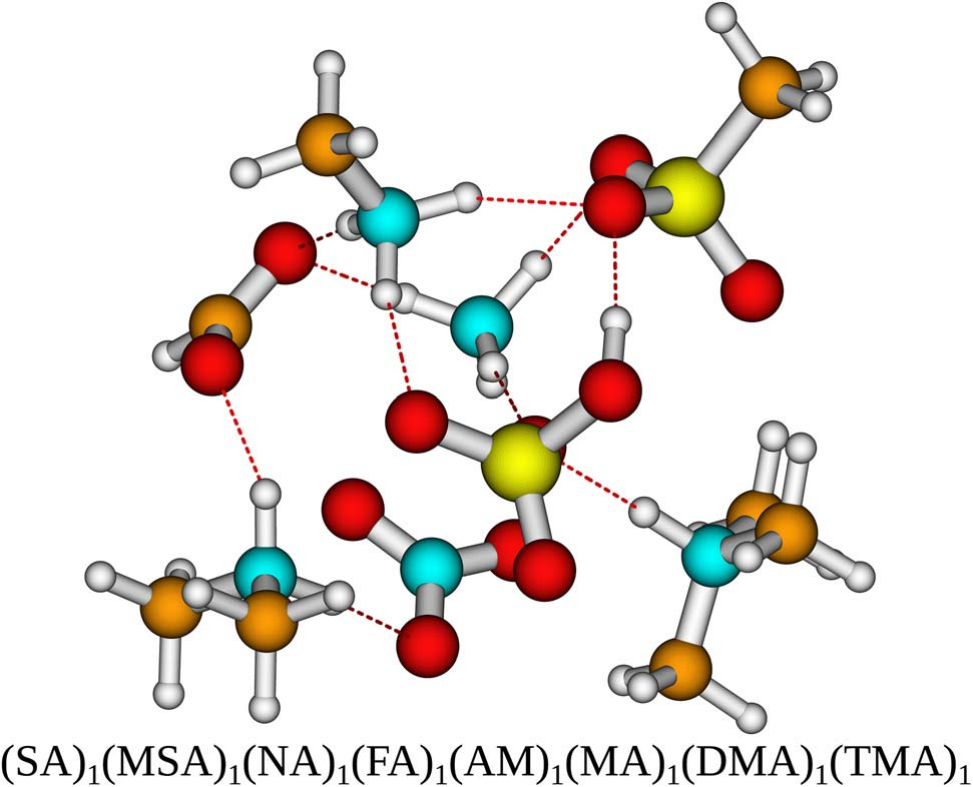
The (SA)_1_(MSA)_1_(NA)_1_(FA)_1_(AM)_1_(MA)_1_(DMA)_1_(TMA)_1_ cluster structure lowest in Gibbs free energy at the ωB97X-D/6-31G++(d,p) level of theory with the quasi-harmonic approximation (cutoff of 100 cm^−1^) for the initial sampling. Yellow = sulfur, red = oxygen, cyan = nitrogen, brown = carbon and white = hydrogen.

To gain a more complete test set we extended the multi-acid–multi-base clusters systems by Knattrup *et al.*^44^ using the same workflow for a total of 288 new AM-containing clusters. With the acids being SA, MSA, FA and NA and the bases being AM, MA, DMA and TMA. This is the first sampling of multi-component clusters containing up to 8 different species yielding a data set where synergistic effects in cluster formation between different species of bases^41^ and acids^44^ and mixes thereof can be studied. Such clusters could be relevant for modeling polluted coastal environments. Fig. 1 presents the molecular structure of a newly sampled 8-component cluster. It is seen that all the acids have transferred a proton to all the bases.

The initial sampling yields binding free energies ranging from −28.43 kcal mol^−1^ [(MSA)_1_(NA)_1_(FA)_1_(AM)_2_] to −104.0 kcal mol^−1^ [(SA)_3_(NA)_1_(AM)_1_(MA)_1_(DMA)_2_] for the cluster configurations lowest in free energy at the ωB97X-D/6-31++G(d,p) level of theory.

### 3.2 Assessment of the AMC-xTB binding energies

**Fig. 2:**
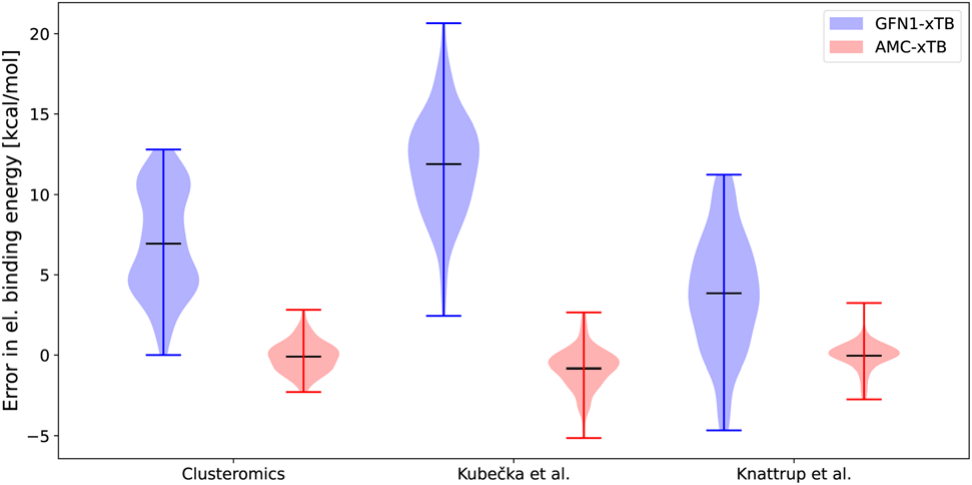
Error in the electronic binding energies for the GFN1-xTB and AMC-xTB methods compared with the ωB97X-D/6-31++G(d,p) level of theory. The clusteromics I–V^48–52^ sets have (SA/FA/MSA/NA)_0–2_(AM/MA/DMA/TMA/EDA)_0–2_ clusters, the Kubečka *et al.*^41^ set has sulfuric acid–multi-base (SA)_1–4_(AM/MA/DMA/TMA/EDA)_1–4_ clusters, Knattrup *et al.*^44^ set has the multi-acid–muti-base (SA/FA/MSA/NA)_1–4_(MA/DMA/TMA)_1–4_ clusters including the new AM-containing clusters sampled in this work.

We reparameterized GFN1-xTB to obtain a new tight-binding semiempirical reparameterization denoted as AMC-xTB. Fig. 2 shows the error in electronic binding energies before (GFN1-xTB) and after (AMC-xTB) reparameterization. The models have been tested on the entire clusteromics I–V^48–52^ data sets (56 436 data points), the sulfuric acid–multi-base (SA)_1–4_(AM/MA/DMA/TMA/EDA)_1–4_ cluster data set (684 data points) by Kubečka *et al.*^41^ and the multi-acid–muti-base (SA/FA/MSA/NA)_1–4_(MA/DMA/TMA)_1–4_ by Knattrup *et al.*^44^ including the new AM-containing clusters (1629 data points). All the tested structures are equilibrium structures at the ωB97X-D/6-31++G(d,p) level of theory. Although the Gaussian version and integration grid used for optimization differ for some structures, it was found to have a negligible effect on this comparison as we are studying the binding energies and not the absolute energies.

For all the data sets shown in Fig. 2 the reparameterization results in near-zero means of the energy errors. This is a reduction from error means of 3.7–11.8 kcal mol^−1^ for GFN1-xTB. In addition, the AMC-xTB model achieves a more narrow error distribution with the root mean square deviations decreasing from 7.6–12.3 kcal mol^−1^ to 0.81–1.45 kcal mol^−1^, implying that there will be fewer outliers. The error span on the larger clusters for the Knattrup *et al.*^44^ and Kubečka *et al.*^41^ sets are similar to the error span for the smaller clusters in the optimization set. This shows that reparameterizing on smaller clusters is adequate for calculations on larger-sized clusters as the model gets some of the underlying chemistry correct and scales effectively with system size. The new AMC-xTB model reduces the number of structures needed to pass from the semiempirical step to the DFT step in configurational sampling. For atmospheric molecular clusters, this implies that the AMC-xTB model is unequivocally better to apply in the configurational sampling funneling workflow compared to GFN1-xTB.

### 3.3 Assessment of the AMC-xTB geometries and gradients

**Fig. 3:**
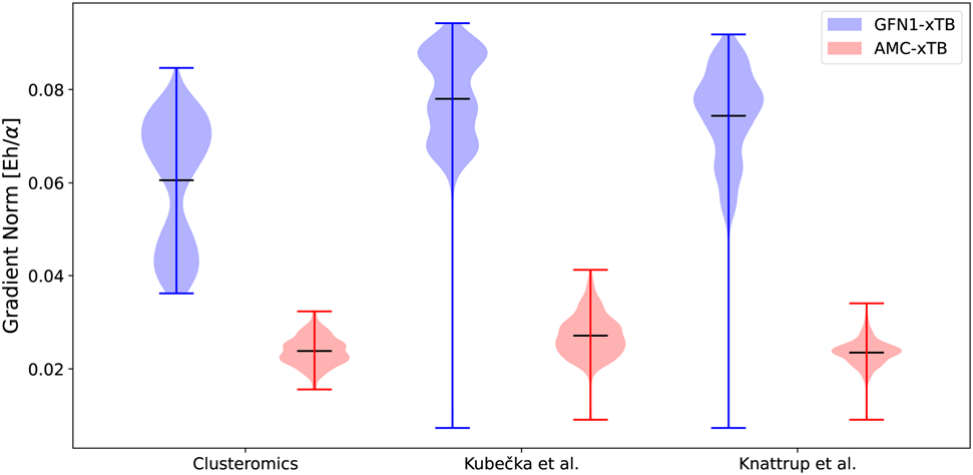
The gradient norms for the GFN1-xTB and AMC-xTB methods. *α* is the Bohr radius. The clusteromics^48–52^ set is (SA/FA/MSA/NA)_0–2_(AM/MA/DMA/TMA/EDA)_0–2_ clusters, the Kubečka *et al.*^41^ set is sulfuric acid–multi-base (SA)_1–4_(AM/MA/DMA/TMA/EDA)_1–4_ clusters, Knattrup *et al.*^44^ is the multi-acid–muti-base (SA/FA/MSA/NA)_1–4_(MA/DMA/TMA)_1–4_ clusters including the new AM-containing clusters sampled in this work. The structures are equilibrium structures at the ωB97X-D/6-31++G(d,p) level of theory.

The gradient norms were also a part of the optimization scheme (eqn (1)). Fig. 3 shows the gradient norms given by the xtb program for the two parameterizations. The structures are equilibrium structures at the ωB97X-D/6-31++G(d,p) level of theory, so the ideal gradient norms should be below the default gradient convergence thresholds of 10^−3^*E*_h_/*α*. None of the methods manages to be below this threshold, but the AMC-xTB model is close. This does not directly mean the model is closer to the correct structure, as the new parameters might just have flattened the potential energy surface at this point without moving closer to the minimum. However, including the gradients in the target function avoids numerical instability, and yields reasonable optimized structures. To test if the structures are closer to a minimum at the DFT level, the initial DFT structures of all three datasets were optimized using the different parameterizations, and the RMSD was computed between the initial DFT structure and the GFN1-xTB/AMC-xTB optimized structures (see Table 1).

**Table 1:** Comparison of the mean, median, and standard deviation (std) of the root-mean-squared differences (RMSD) between the initial DFT structure and the optimized structure at the given parameterization. The clusteromics I–V^48–52^ sets includes the (SA/FA/MSA/NA)_0–2_(AM/MA/DMA/TMA/EDA)_0–2_ clusters, the Kubečka *et al.*^41^ set comprise the sulfuric acid–multi-base (SA)_1–4_(AM/MA/DMA/TMA/EDA)_1–4_ clusters and Knattrup *et al.*^44^ set has the multi-acid–muti-base (SA/FA/MSA/NA)_1–4_(MA/DMA/TMA)_1–4_ clusters including the new AM-containing clusters sampled in this work. The lowest errors are shown in bold

Method/data set	Mean	Median	Std
GFN1-xTB/clusteromics	0.484	0.387	0.330
AMC-xTB/clusteromics	**0.355**	**0.242**	**0.282**
GFN1-xTB/Knattrup *et al.*	0.378	0.367	0.138
AMC-xTB/Knattrup *et al.*	**0.235**	**0.193**	**0.125**
GFN1-xTB/Kubečka *et al.*	0.376	0.361	0.140
AMC-xTB/Kubečka *et al.*	**0.189**	**0.164**	**0.094**

We find that the AMC-xTB model reduces the mean RMSD of the full clusteromics set from 0.484 Å to 0.355 Å and a similar reduction is seen for the Knattrup *et al.*^44^ and Kubečka *et al.*^41^ sets with RMSDs being reduced from 0.378 Å to 0.235 Å and from 0.376 Å to 0.189 Å, respectively. This, coupled with the smaller gradients, suggests that the reparameterized model is closer to a minimum at the DFT level. This implies that the preoptimization step in a funneling approach with the AMC-xTB model compared to GFN1-xTB yields structures closer to the DFT structure and will likely reduce the subsequent optimization time at the DFT level.

### 3.4 Test of new configurational sampling workflows

To further test how the new AMC-xTB model fares in cluster configurational sampling, we tested the independent and improvement workflow for several previously studied (acid)_4_(base)_4_ cluster systems. Hence, the workflow is tested on clusters up to twice the size of those used in the reparameterization.

**Fig. 4:**
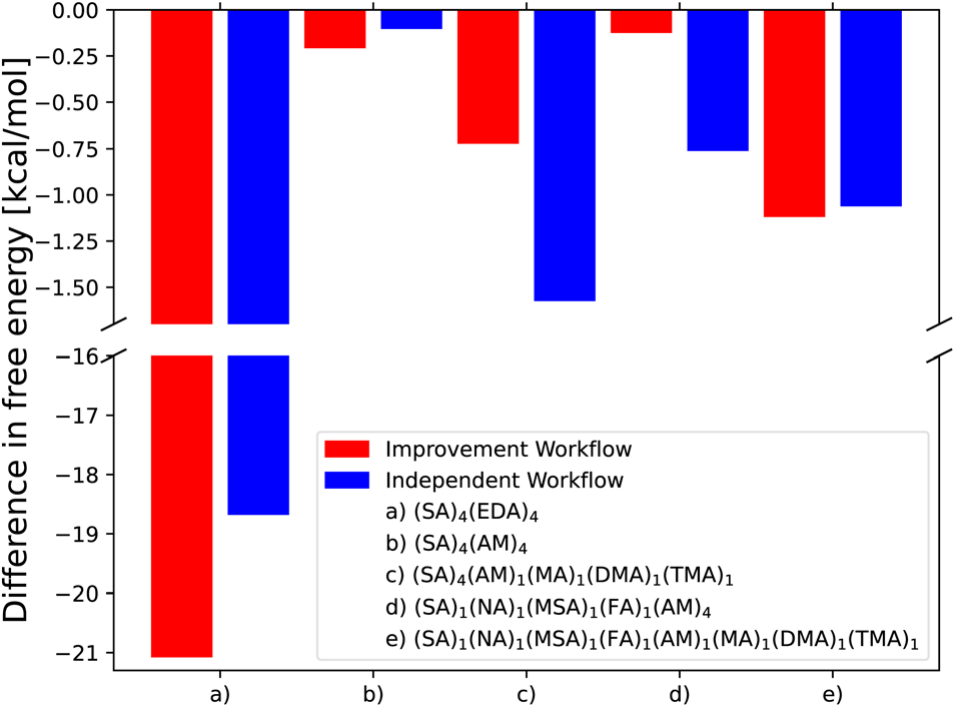
Comparison of the lowest free energy conformer found by the independent and improvement configurational workflows compared to the configurations found by Kubečka *et al.*^41^ (a–c) and two new multi-component AM clusters sampled in the same way as Knattrup *et al.*^44^ (d and e). Gibbs free energies are calculated at the ωB97X-D/6-31++G(d,p) level of theory with the quasi-harmonic approximation (cutoff of 100 cm^−1^) and vib. frequencies scaled by 0.996 in accordance with Kubečka *et al.*^41^

Fig. 4 shows the difference in binding free energy for the lowest free-energy configuration found by employing the independent and improvement workflows for the (SA)_4_(EDA)_4_, (SA)_4_(AM)_4_, and (SA)_4_(AM)_1_(MA)_1_(DMA)_1_(TMA)_1_ clusters compared to Kubečka *et al.*^41^ and the (SA)_1_(MSA)_1_(NA)_1_(FA)_1_(AM)_4_ and the new (SA)_1_(MSA)_1_(NA)_1_(FA)_1_(AM)_1_(MA)_1_(DMA)_1_(TMA)_1_ clusters sampled in this work. The SA–AM clusters have been extensively studied previously^6,56–61^ and are therefore believed to be well-sampled using the original configurational sampling procedure and thereby difficult to improve. Still, the new CREST + AMC-xTB methodology manages to find cluster structures lower in free energy by 0.21 kcal mol^−1^ compared to the previous works.

**Fig. 5:**
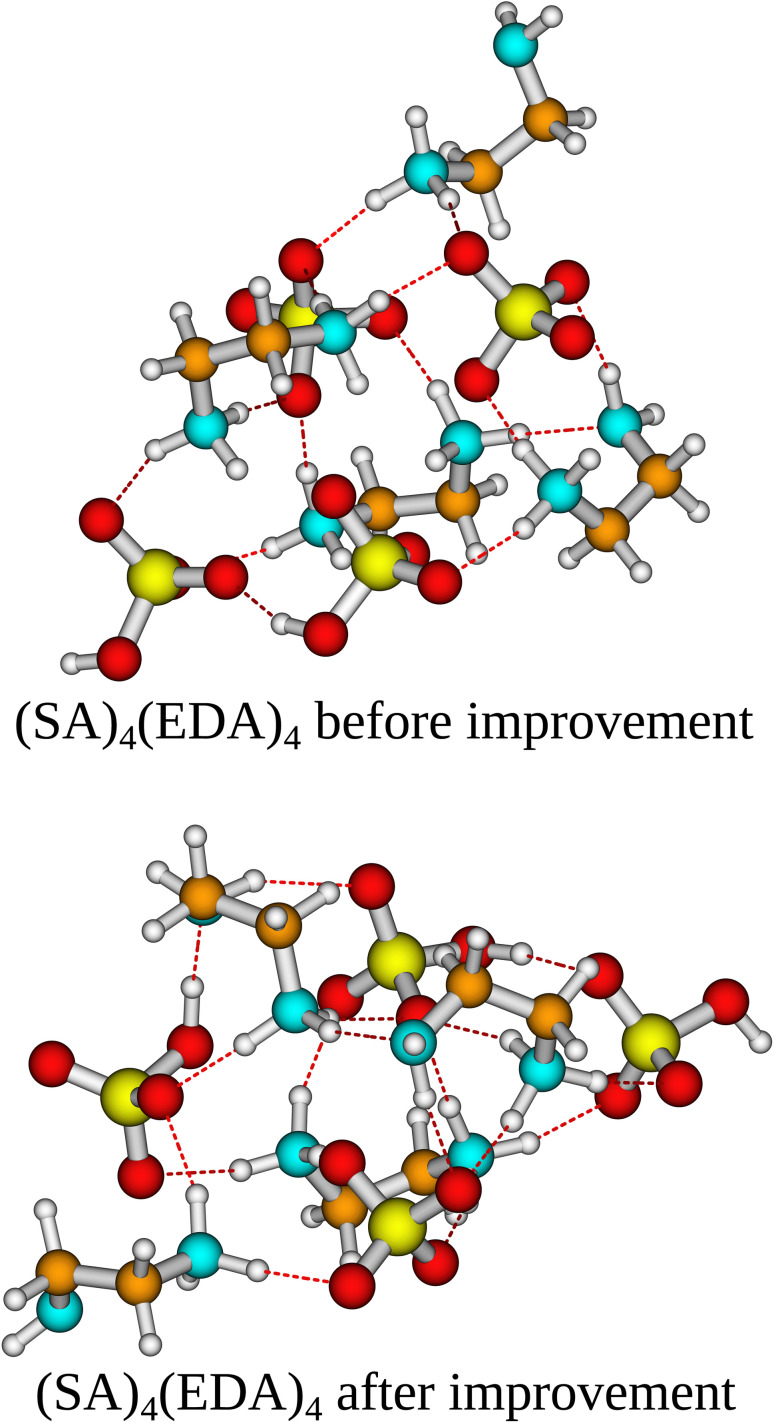
The (SA)_4_(EDA)_4_ cluster structure lowest in Gibbs free energy at the ωB97X-D/6-31G++(d,p) level of theory with the quasi-harmonic approximation (cutoff of 100 cm^−1^) before and after the improvement workflow. Yellow = sulfur, red = oxygen, cyan = nitrogen, brown = carbon, and white = hydrogen.

In the case of the (SA)_4_(AM)_4_ and (SA)_1_(MSA)_1_(NA)_1_(FA)_1_(AM)_1_(MA)_1_(DMA)_1_(TMA)_1_ clusters, the independent/improvement workflows perform similar and yield similar free energy improvements. However, for the (SA)_4_(AM)_1_(MA)_1_(DMA)_1_(TMA)_1_ clusters, the independent workflow works slightly better, finding a cluster 0.85 kcal mol^−1^ lower in free energy compared to the improvement workflow. This illustrates that the sampling is very sensitive to the configuration used as input for CREST, although it might also be due to the randomness of the dynamic processes in CREST. The reason for the difference might be that the original work's configurational sampling was worse than the independent workflow, yielding a worse starting structure for the CREST sampling within the improvement workflow. We see a massive improvement in the configurational sampling of the (SA)_4_(EDA)_4_ clusters by −18/−21 kcal mol^−1^. This is caused by the flexibility of the EDA molecule, as it is the only monomer that contains a C–C bond it can rotate around, making it difficult to sample the full configurational space using only ABCluster with rigid molecules. This improvement should primarily be attributed to the inclusion of metadynamics sampling in CREST and not purely the parameterization of AMC-xTB as it allows the EDA to rotate around its bonds and find a structure with more/better paired intermolecular interactions as seen in Fig. 5. It should also be noted that the main improvements are the electronic binding energy and the thermal contribution varies very little between the clusters.

However, this shows the strength of the presented workflows as they can be used for clusters containing more flexible organic molecules.

### 3.5 Massive improvement test

**Fig. 6:**
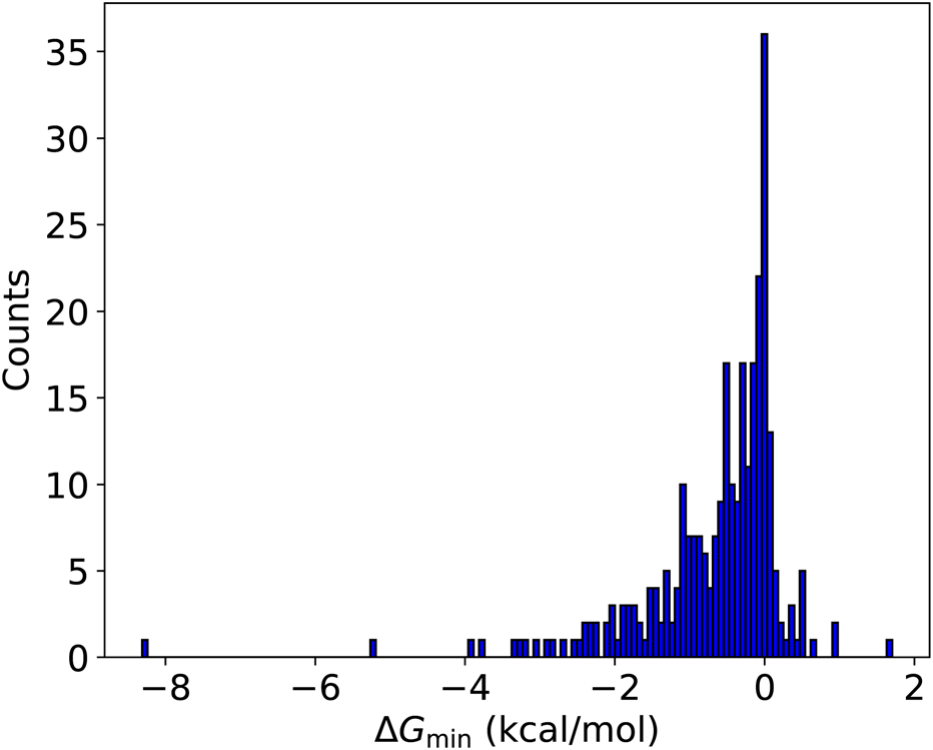
Comparison of the lowest free energy conformer found by the improvement configurational workflow compared to the original workflow. Gibbs free energies are calculated at the ωB97X-D/6-31++G(d,p) level of theory with the quasi-harmonic approximation (cutoff of 100 cm^−1^).

Based on the previous sections, it is clear that the improvement workflow could locate more stable clusters. As the potential energy surface of multi-acid–multi-base clusters becomes very complicated, here we test this new approach for such systems. These 288 new AM-containing clusters were used as a massive test set for the improvement workflow using the newly parameterized AMC-xTB model. The improvement workflow manages to find configurations lower in free energy at the ωB97X-D/6-31++G(d,p) for 245 out of the 288 clusters (85.1%) as can be seen in Fig. 6.

In most cases, the improvement is between 0–2 kcal mol^−1^. However, for the (SA)_1_(MSA)_1_(NA)_1_(FA)_1_(AM)_1_(DMA)_2_(TMA)_1_, (SA)_3_(NA)_1_(AM)_1_(MA)_3_, and (SA)_3_(FA)_1_(AM)_1_(MA)_1_(DMA)_2_ clusters a massive improvement of 8.3, 5.2 and 3.9 kcal mol^−1^ is observed, respectively.

Comparing the conformer index at the AMC-xTB level of theory with the final ωB97X-D/6-31++G(d,p) level of theory with the quasi-harmonic approximation (cutoff of 100 cm^−1^) the Gibbs free energy minimum at the DFT level is also the electronic energy minimum at the AMC-xTB level of theory for 66 of the clusters (see Fig. S1†). If 10 conformers are included from the AMC-xTB level of theory, the free energy minimum energy is captured for 155 out of the 288 clusters with improvements found for 209 (see Fig. S2†). Furthermore, the maximum error is 2 kcal mol^−1^ with a mean of 0.12 kcal mol^−1^ when reducing from 50 to 10 conformers.

This highlights the need for including dynamics-based sampling procedures for atmospheric clusters even though the system might seem fairly rigid. It can also be envisioned that the improvement workflow will be quite important when studying much larger (SA)_1–20_(base)_1–20_ clusters as recently done by Engsvang *et al.*^42,62^ and Wu *et al.*^38^ For large clusters, the global minimum is tricky to locate, and adding dynamics-based configurational sampling might aid in the process.

## 4 Conclusions

We have reparameterized the GFN1-xTB model to yield better binding electronic energies and gradient norms for atmospherically relevant clusters composed of the following species: sulfuric acid (SA), methanesulfonic acid (MSA), nitric acid (NA), formic acid (FA), ammonia (AM), methylamine (MA), dimethylamine (DMA), trimethylamine (TMA), and ethylenediamine (EDA). The reparameterization, denoted AMC-xTB, for use in the xtb/CREST program, is based on the ωB97X-D/6-31++G(d,p) level of theory. The model shows a substantial decrease in the error of the binding electronic energies compared to the original GFN1-xTB parameterization and the gradient norms of the equilibrium structures are closer ωB97X-D/6-31++G(d,p) level of theory compared to GFN1-xTB. The reparameterization strategy is general and can be used to reparameterize other methods such as GFN2-xTB.

We tested two new configurational sampling procedures with the new parameterizations being employed in the xTB and CREST programs. The first workflow, denoted as “improvement workflow,” is based on improving the best structure currently known in the literature with CREST and then doing the DFT calculations. The second workflow, denoted the “independent workflow,” starts by configurational sampling using ABCluster, followed by xtb, CREST, and then DFT. Using the two workflows, we find cluster structures lower in free energy for the following (SA)_4_(EDA)_4_, (SA)_4_(AM)_4_,(SA)_4_(AM)_1_(MA)_1_(DMA)_1_(TMA)_1_, (SA)_1_(MSA)_1_(NA)_1_(FA)_1_(AM)_4_ and (SA)_1_(MSA)_1_(NA)_1_(FA)_1_(AM)_1_(MA)_1_(DMA)_1_(TMA)_1_ systems in all cases compared to the best-known value in the literature.

Testing the improvement workflow on 288 large multi-acid–multi-base cluster systems, the workflow finds improvements for 85.1% of the clusters, showing the need for dynamics-based sampling.

The parameterization strategy given here is not specific to either GFN1-xTB or atmospheric clusters and could be used in general. For instance, one could imagine increasing the number of d-functions in the basis set for sulfur atoms in GFN2-xTB and then reparameterizing the new GFN2-xTB model or doing a reparameterization for much larger clusters.

## Author contributions

Conceptualization: J. E.; methodology: Y. K., J. K., H. W., F. J. and J. E.; software: F. J.; formal analysis: Y. K. and J. K.; investigation: Y. K., J. K. and H. W.; resources: J. E.; writing – original draft: Y. K., J. K. and J. E.; writing – review & editing: Y. K., J. K., H. W., F. J. and J. E.; visualization: Y. K.; project administration: J. E.; funding acquisition: J. E.; supervision: F. J. and J. E.

## Conflicts of interest

There are no conflicts to declare.

## Supplementary Material

RA-014-D4RA03021D-s001

## References

[cit1] Canadell J. G. , Monteiro P. M. S., Costa M. H., Cotrim da Cunha L., Cox P., Eliseev A. V., Henson S., Ishii M., Jaccard S., Koven C. and et al., in Global Carbon and Other Biogeochemical Cycles and Feedbacks, ed. V. Masson-Delmotte, P. Zhai, A. Pirani, S. L. Connors, C. Péan, S. Berger, N. Caud, Y. Chen, L. Goldfarb, M. I. Gomis and et al., Cambridge University Press, Cambridge, United Kingdom and New York, NY, USA, 2021, pp. 673–816

[cit2] Boucher O., Lohmann U. (1995). Tellus B.

[cit3] Merikanto J., Spracklen D. V., Mann G. W., Pickering S. J., Carslaw K. S. (2009). Atmos. Chem. Phys..

[cit4] Almeida J., Schobesberger S., Kürten A., Ortega I. K., Kupiainen-Määttä O., Praplan A. P., Adamov A., Amorim A., Bianchi F., Breitenlechner M. (2013). et al.. Nature.

[cit5] Elm J., Kubečka J., Besel V., Jääskeläinen M. J., Halonen R., Kurtén T., Vehkamäki H. (2020). J. Aerosol Sci..

[cit6] Engsvang M., Wu H., Knattrup Y., Kubečka J., Jensen A. B., Elm J. (2023). Chem. Phys. Rev..

[cit7] Elm J., Ayoubi D., Engsvang M., Jensen A. B., Knattrup Y., Kubečka J., Bready C. J., Fowler V. R., Harold S. E., Longsworth O. M. (2023). *et al.*. Wiley Interdiscip. Rev.: Comput. Mol. Sci..

[cit8] Jokinen T., Sipilä M., Junninen H., Ehn M., Lönn G., Hakala J., Petäjä T., Mauldin R. L. I., Kulmala M., Worsnop D. R. (2012). Atmos. Chem. Phys..

[cit9] Zapadinsky E., Passananti M., Myllys N., Kurtén T., Vehkamäki H. (2019). J. Phys. Chem. A.

[cit10] Passananti M., Zapadinsky E., Zanca T., Kangasluoma J., Myllys N., Rissanen M. P., Kurtén T., Ehn M., Attoui M., Vehkamäki H. (2019). Chem. Commun..

[cit11] Lehtipalo K., Yan C., Dada L., Bianchi F., Xiao M., Wagner R., Stolzenburg D., Ahonen L. R., Amorim A., Baccarini A. (2018). et al.. Sci. Adv..

[cit12] Pracht P., Grimme S., Bannwarth C., Bohle F., Ehlert S., Feldmann G., Gorges J., Müller M., Neudecker T., Plett C., Spicher S., Steinbach P., Wesołowski P. A., Zeller F. (2024). J. Chem. Phys..

[cit13] Pracht P., Bohle F., Grimme S. (2020). Phys. Chem. Chem. Phys..

[cit14] Dieterich J. M., Hartke B. (2010). Mol. Phys..

[cit15] Zhang J., Dolg M. (2015). Phys. Chem. Chem. Phys..

[cit16] Zhang J., Dolg M. (2016). Phys. Chem. Chem. Phys..

[cit17] Kubečka J., Besel V., Kurtén T., Myllys N., Vehkamäki H. (2019). J. Phys. Chem. A.

[cit18] Hou G.-L., Zhang J., Valiev M., Wang X.-B. (2017). Phys. Chem. Chem. Phys..

[cit19] Li H., Kupiainen-Määttä O., Zhang H., Zhang X., Ge M. (2017). Atmos. Environ..

[cit20] Ling J., Ding X., Li Z., Yang J. (2017). J. Phys. Chem. A.

[cit21] Zhang H., Kupiainen-Määttä O., Zhang X., Molinero V., Zhang Y., Li Z. (2017). J. Chem. Phys..

[cit22] Odbadrakh T. T., Gale A. G., Ball B. T., Temelso B., Shields G. C. (2020). JoVE.

[cit23] Ball B. T., Vanovac S., Odbadrakh T. T., Shields G. C. (2021). J. Phys. Chem. A.

[cit24] Harold S. E., Bready C. J., Juechter L. A., Kurfman L. A., Vanovac S., Fowler V. R., Mazaleski G. E., Odbadrakh T. T., Shields G. C. (2022). J. Phys. Chem. A.

[cit25] Bready C. J., Vanovac S., Odbadrakh T. T., Shields G. C. (2022). J. Phys. Chem. A.

[cit26] Longsworth O. M., Bready C. J., Shields G. C. (2023). Environ. Sci.: Atmos..

[cit27] Longsworth O. M., Bready C. J., Joines M. S., Shields G. C. (2023). Environ. Sci.: Atmos..

[cit28] Wales D. J., Doye J. P. K. (1997). J. Phys. Chem. A.

[cit29] Zhu Y.-P., Liu Y.-R., Huang T., Jiang S., Xu K.-M., Wen H., Zhang W.-J., Huang W. (2014). J. Phys. Chem. A.

[cit30] Peng X.-Q., Liu Y.-R., Huang T., Jiang S., Huang W. (2015). Phys. Chem. Chem. Phys..

[cit31] Miao S.-K., Jiang S., Chen J., Ma Y., Zhu Y.-P., Wen Y., Zhang M.-M., Huang W. (2015). RSC Adv..

[cit32] Lv S.-S., Miao S.-K., Ma Y., Zhang M.-M., Wen Y., Wang C.-Y., Zhu Y.-P., Huang W. (2015). J. Phys. Chem. A.

[cit33] Grimme S., Bannwarth C., Shushkov P. (2017). J. Chem. Theory Comput..

[cit34] Bannwarth C., Ehlert S., Grimme S. (2019). J. Chem. Theory Comput..

[cit35] Stewart J. J. P. (2007). J. Mol. Model..

[cit36] Stewart J. J. P. (2013). J. Mol. Model..

[cit37] Jensen A. B., Kubečka J., Schmitz G., Christiansen O., Elm J. (2022). J. Chem. Theory Comput..

[cit38] Wu H., Engsvang M., Knattrup Y., Kubečka J., Elm J. (2023). ACS Omega.

[cit39] Knattrup Y., Kubečka J., Ayoubi D., Elm J. (2023). ACS Omega.

[cit40] Kubečka J., Rasmussen F. R., Christensen A. S., Elm J. (2022). Environ. Sci. Technol. Lett..

[cit41] Kubečka J., Neefjes I., Besel V., Qiao F., Xie H.-B., Elm J. (2023). J. Phys. Chem. A.

[cit42] Engsvang M., Kubečka J., Elm J. (2023). ACS Omega.

[cit43] Kubečka J., Knattrup Y., Engsvang M., Jensen A. B., Ayoubi D., Wu H., Christiansen O., Elm J. (2023). Nat. Comput. Sci..

[cit44] Knattrup Y., Kubečka J., Elm J. (2023). J. Phys. Chem. A.

[cit45] Temelso B., Mabey J. M., Kubota T., Appiah-Padi N., Shields G. C. (2017). J. Chem. Inf. Model..

[cit46] Kubečka J., Besel V., Neefjes I., Knattrup Y., Kurtén T., Vehkamäki H., Elm J. (2023). ACS Omega.

[cit47] Frisch M. J. , Trucks G. W., Schlegel H. B., Scuseria G. E., Robb M. A., Cheeseman J. R., Scalmani G., Barone V., Petersson G. A., Nakatsuji H. and *et al.*, Gaussian 16, Revision A 03, Gaussian, Inc., Wallingford CT, 2016

[cit48] Elm J. (2021). ACS Omega.

[cit49] Elm J. (2021). ACS Omega.

[cit50] Elm J. (2022). ACS Omega.

[cit51] Knattrup Y., Elm J. (2022). ACS Omega.

[cit52] Ayoubi D., Knattrup Y., Elm J. (2023). ACS Omega.

[cit53] Myllys N., Elm J., Kurtén T. (2016). Comput. Theor. Chem..

[cit54] Elm J. (2019). ACS Omega.

[cit55] Jensen F. (2001). J. Chem. Phys..

[cit56] Besel V., Kubečka J., Kurtén T., Vehkamäki H. (2020). J. Phys. Chem. A.

[cit57] Leverentz H. R., Siepmann J. I., Truhlar D. G., Loukonen V., Vehkamäki H. (2013). J. Phys. Chem. A.

[cit58] Ortega I. K., Kupiainen O., Kurtén T., Olenius T., Wilkman O., McGrath M. J., Loukonen V., Vehkamäki H. (2012). Atmos. Chem. Phys..

[cit59] Myllys N., Kubečka J., Besel V., Alfaouri D., Olenius T., Smith J. N., Passananti M. (2019). Atmos. Chem. Phys..

[cit60] Nadykto A. B., Yu F. (2007). Chem. Phys. Lett..

[cit61] Kurtén T., Torpo L., Sundberg M. R., Kerminen V., Vehkamäki H., Kulmala M. (2007). Atmos. Chem. Phys..

[cit62] Engsvang M., Elm J. (2022). ACS Omega.

