# Supplementary material for: Reparameterization of GFN1-xTB for atmospheric molecular clusters: applications to multi-acid–multi-base systems
Source: RSC Adv. 2024 Jun 21;14(28):20048–55. doi: 10.1039/d4ra03021d (PMC11191700; doi:10.1039/d4ra03021d)
Supplement: RA-014-D4RA03021D-s001 [file RA-014-D4RA03021D-s001.pdf]

Electronic Supplementary Material (ESI) for RSC Advances.
This journal is © The Royal Society of Chemistry 2024

Supporting Information for Reparameterization of GFN1-xTB for Atmospheric Molecular Clusters: Applications to Multi-Acid–Multi-Base Systems

Yosef Knattrup, Jakub Kubečka, Haide Wu, Frank Jensen, and Jonas Elm*

Department of Chemistry, Aarhus University, Langelandsgade 140, 8000 Aarhus C, Denmark

E-mail: jelm@chem.au.dk

Phone: +45 28938085

1 Massive Improvement Test SI Figures

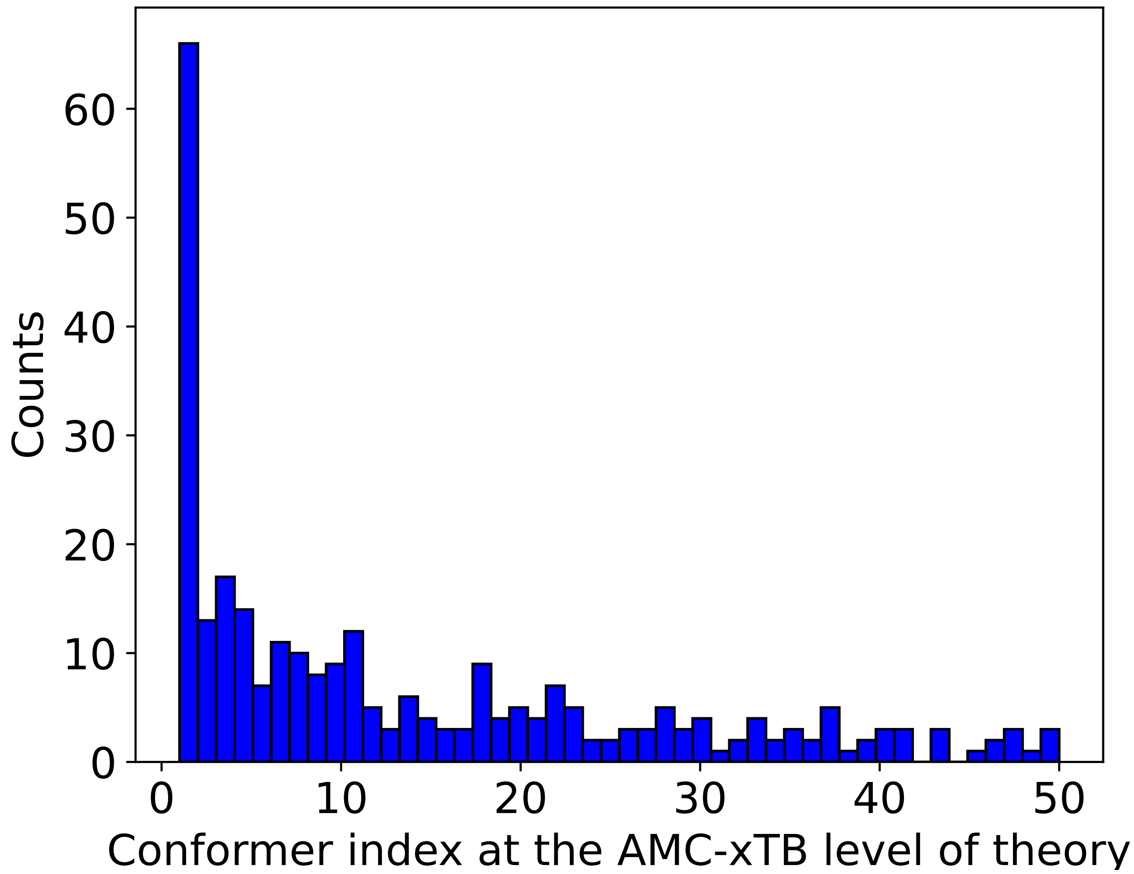

Figure 1: Conformer index at the AMC-xTB level of theory out of the 50 conformers optimized at the ωB97X-D/6-31++G(d,p) level of theory with the quasi-harmonic approximation (cutoff of 100 cm^{-1}) that end up being the Gibbs free minimum.

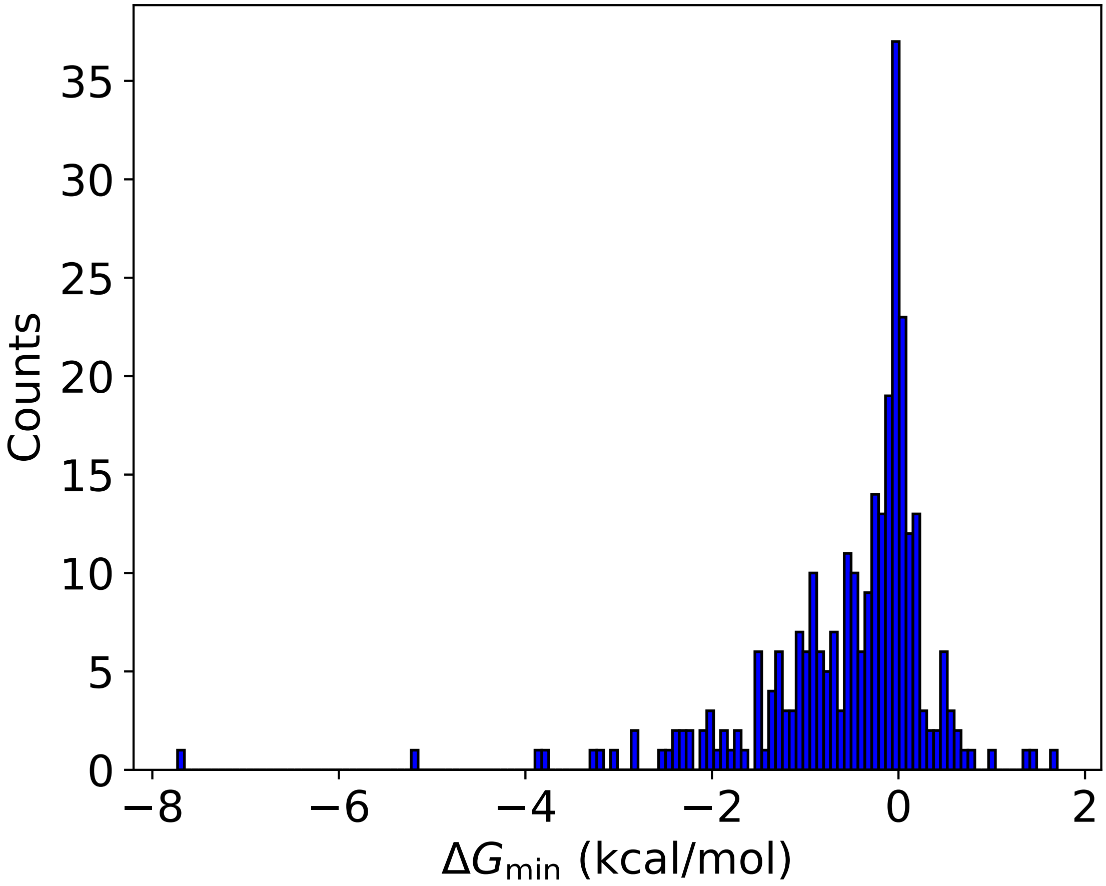

Figure 2: Comparison of the lowest free energy conformer found by the improvement configurational workflow compared to the original workflow if only 10 conformers are selected for DFT optimization. Calculated at the ωB97X-D/6-31++G(d,p) level of theory with the quasi-harmonic approximation (cutoff of 100 cm^{-1}).

2 Parameter file and cluster structures/properties

The parameter file is available in the Atmospheric Cluster DataBase (ACDB) at: `https://github.com/elmjonas/ACDB/tree/master/Articles/knattrup24_am-clusters_amc-xtb/Additional_files/`

The parameter file needs to be in the directory where the calculation is occurring OR in the directory the *xtbpath* environmental variable is pointed at. For CREST, the parameter file needs to be in the directory set by the environmental variable as CREST creates several subfolders during its calculations.

All the structures (xyz format) and properties are available in ACDB at: `https://github.com/elmjonas/ACDB/tree/master/Articles/knattrup24_am-clusters_amc-xtb/`

See here how to handle the pickle file format: `https://github.com/elmjonas/ACDB/tree/master?tab=readme-ov-file#using-the-pickled-files`
